# Current Hospital Topics

**Published:** 1906-08-11

**Authors:** 


### HOSPITAL ADMINISTRATION.

### CONSTRUCTION AND ECONOMICS.

### CURRENT HOSPITAL TOPICS.

#### The Buffalo Hospital Conference.

The eighth annual Conference of the Association of Hospital Superintendents of the United States will be held in Buffalo from September 18 to 21 next. The Conference will be welcomed to Buffalo by the Mayor of the city, and elaborate preparations will be made to make the proceedings helpful as well as enjoyable. The programme includes papers on practical subjects of hospital management, and provides ample opportunity for full discussion and the interchange of experiences in regard to hospital administration. During the current year this Association has considerably developed, and the results of the Boston Conference have made their influence felt for good throughout the American hospital world. The headquarters of the Association at Buffalo will be the Niagara Hotel, and all information can be obtained from the President, Mr. Geo. P. Ludlam, New York City, or from Mr. Geo. Bailey, junior, Philadelphia, the Secretary of the Conference.

#### Sir Sydney Waterlow.

The death of Sir Sydney Waterlow has removed a familiar figure from the Council of the Metropolitan Hospital Sunday Fund. The present writer supplied Sir Sydney Waterlow with the material and information on which the Metropolitan Hospital Sunday Fund was established in his Lord Mayoralty. During the thirty-four intervening years he has been associated with Sir Sydney in hospital affairs, and he is able to state from intimate knowledge that there are probably very few who have the cause of the hospitals closer to their hearts than had Sir Sydney This spirit found expression at Cannes, which owes its present excellent hospital provision for English residents to the inspiration and zeal of the late Sir Sydney Waterlow. The Sunday Fund was, of course, to Sir Sydney a great pride and pleasure. He presided over the Distribution Committee for upwards of a quarter of a century, and the fact that the proceedings of that committee have commended themselves to the judgment and acceptance of all classes of opinion is probably the highest testimony to the wisdom and fairness of its proceedings which any public man can place to his record. During the last few years Sir Sydney's health had prevented him from taking a part in hospital affairs, but his interest in them never flagged, and during the spring of the present year he was actively discussing the intricacies of the out-patient question and collecting data with a view to issuing a manifesto on the subject. In tendering our condolences to Lady Waterlow and the members of Sir Sydney's family we can assure them that they have at this time the sympathy of many hospital workers, especially in London.

#### Pain the Price of Progress.

The Bishop of Sheffield in a recent sermon laid down the doctrine that the price we had to pay for progress was the price of pain, and the world of progress would be much worse if it were not for the softening influences of pain and suffering. The Bishop strongly insisted that the pathway of pleasure was the pathway of retrogression and defeat, and that the strenuous life was the only life worthy of the people of a nation which professed Christianity. Dealing with the question of putting hospitals on the rates, the Bishop asked what was to be said of such a cowardly avoidance of a plain duty, when only recently the gate-money at a football match was £5,000? That the English people could find abundance of money for their pleasures was proved by the fact that the amount paid to professional footballers in England to-day was £53,000 a year, as compared with £3,500 only ten years ago. A multitude of people felt no trouble was too great to secure excessive indulgence in pleasures, but in commercial matters the prize would go to that country where the people were not afraid of pain. He made an eloquent appeal to the English people to consider the huge differences which existed in the amounts at present given by some Englishmen and Englishwomen to pleasure and to pain. He appealed to them not to spend such large sums on pleasure as a consequence of not doing their duty to the relief of pain. The Bishop made a good point here in his plea for the hospitals of Sheffield, and, indeed, for all hospitals. The principle of personal service in the days of health in the cause of the sick when loyally pursued is the best recognition of the claims which freedom from pain imposes upon the healthy. The League of Mercy has taught a multitude of people to set themselves seriously to the endeavour to acquit themselves like men and women in the days of health in the cause of the sick. Could not the Bishop of Sheffield usefully introduce a similar movement into Sheffield and throughout his diocese?

#### Hospitals in East London.

It is not sufficiently appreciated that on the east side the distance of the boundary of the outer ring and Greater London from Charing Cross is much less than it is on the north, west, and south sides of the area in question. The importance of remembering this lies mainly in the fact that the residents of the outer ring in the east are brought into closer touch with those Metropolitan hospitals which minister to the requirements of the dense working population which resides within comparatively a short distance of the London and Poplar Hospitals. There has been a tendency of late to argue that the circumstances of the London and Poplar Hospitals are identical with the circumstances of most Metropolitan hospitals in regard to out-patient relief especially. This is not the case in fact, however, for the residents in the outer ring on the north, south, and west sides of Greater London obtain most, if not all, their out-patient relief from institutions situated in the neighbourhood where they habitually reside. It is not justifiable to state, in the sense in which the words appear to be used, that if there were other hospitals in East London the number of patients in the London Hospital would not be so great as at present. In fact, there is a deep and growing desire in the city of London to help forward the extension and full development of the West Ham and East London Hospital, because it is felt that the development and considerable extension of this institution are urgently demanded in the best interests of the patients and the public. The reason why the London Hospital has so few competitors in East London is, as we have said, that on the east side of Greater London the distance of the boundary of the outer ring from Charing Cross is much less than on any other side. It is perfectly clear that the London Hospital cannot be extended beyond its present limits, which have attained the maximum, and any extension in regard to hospitals which may be called for to supply the needs of the poor of East London must come in the first instance through the West Ham Hospital. Hence the growing and extending interest in the development of that institution.

### The Hospitals and the Sunday Fund.

We have never had any sympathy with the financial mismanagement of any hospital, and are fully alive to the mistakes which have been made in the past in connection with Charing Cross Hospital. At the same time we regard with grave apprehension a new departure made by the Hospital Sunday Fund this year in withholding the award from Charing Cross Hospital until it has proved to the satisfaction of the Distribution Committee that the result of a special appeal to be issued in October has enabled the Committee to carry on the work of this hospital. In the appeals and papers which have been circulated this year in support of Hospital Sunday in London a great point has been made of the fact that within a few weeks of Hospital Sunday every penny raised will be divided among the hospitals "for their maintenance only, to keep wards open, and enable beds to be tenanted. Not a penny for building or for bricks and mortar." The public were told that the money which came from them would this year go direct and immediately to the patients. On this basis the whole of the sum collected has been raised. Further, for thirty-three years the managers of every participating charity have received a cheque for the amount of the Sunday Fund grant at this season of the year, and amongst them Charing Cross Hospital. The principle of the Sunday Fund is that the money collected shall be expended in the year during which it is given for the benefit of the sick of the Metropolis. Charing Cross Hospital finds itself in a very tight place financially at the moment, and that tightness has been made extraordinarily acute by the withholding of the £1,200 given each year at this season, which the managers had every right to look upon as assured income. If the treatment extended to Charing Cross Hospital is to prevail in future in regard to many of the grants of the Hospital Sunday Fund, more than half its usefulness to the hospitals will be removed, and the popularity and financial success of the Fund must, in our judgment, suffer materially. In a nutshell Charing Cross Hospital requires £500 a month from outside sources to keep its beds open—*i.e.* its assured income is roughly £12,000 a year, and its expenditure on the present reformed basis £18,000 a year. It is not business to meet the patriotic endeavours of Lord Kilmorey and other independent gentlemen who have come forward in the public interest on behalf of Charing Cross Hospital by suddenly withholding supplies. It is bad business, too, to cripple and embarrass Lord Kilmorey initially by withholding money which, according to the wording of the appeal to the public on Hospital Sunday and the practice of thirty-three years, hospital managers are entitled to consider to be assured income which will be available in the month of August to help them over present financial difficulties pending the issue of an autumn appeal.

